# Higher Work-Privacy Conflict and Lower Job Satisfaction in GP Leaders and Practice Assistants Working Full-Time Compared to Part-Time: Results of the IMPROVE*job* Study

**DOI:** 10.3390/ijerph19052618

**Published:** 2022-02-24

**Authors:** Julian Göbel, Manuela Schmidt, Tanja Seifried-Dübon, Karen Linden, Lukas Degen, Esther Rind, Anna-Lisa Eilerts, Claudia Pieper, Matthias Grot, Brigitte Werners, Verena Schröder, Karl-Heinz Jöckel, Monika A. Rieger, Birgitta M. Weltermann, on behalf of the IMPROVEjob Consortium

**Affiliations:** 1 Institute of General Practice and Family Medicine, University Hospital Bonn, University of Bonn, Germany Venusberg-Campus 1, 53127 Bonn, Germany; manuela.schmidt@ukbonn.de (M.S.); karen.linden@ukbonn.de (K.L.); lukas.degen@ukbonn.de (L.D.); birgitta.weltermann@ukbonn.de (B.M.W.); 2 Department of Psychosomatic Medicine and Psychotherapy, University Hospital Tuebingen, Osianderstraße 5, 72076 Tuebingen, Germany; tanja.seifried@med.uni-tuebingen.de; 3 Institute of Occupational and Social Medicine and Health Services Research, University Hospital Tuebingen, Wilhelmstr. 27, 72074 Tuebingen, Germany; esther.rind@med.uni-tuebingen.de (E.R.); monika.rieger@med.uni-tuebingen.de (M.A.R.); 4 Institute for Medical Informatics, Biometry and Epidemiology, University Hospital Essen, Hufelandstr. 55, 45147 Essen, Germany; anna-lisa.eilerts@uk-essen.de (A.-L.E.); claudia.pieper@uk-essen.de (C.P.); 5 Institute of Management, Operations Research, Ruhr University Bochum, Universitätsstr. 150, 44801 Bochum, Germany; matthias.grot@rub.de (M.G.); or@rub.de (B.W.); 6 Center for Clinical Trials, University Hospital Essen, Hufelandstr. 55, 45147 Essen, Germany; verena.schroeder@uk-essen.de (V.S.); k-h.joeckel@uk-essen.de (K.-H.J.)

**Keywords:** work-privacy conflict, job satisfaction, general practitioner, general practice leader, practice assistant, primary care, working conditions

## Abstract

Background: Work-privacy conflict (WPC) has become an important issue for medical professionals. The cluster-randomized controlled IMPROVE*job* study aimed at improving job satisfaction (primary outcome), with additional outcomes such as examining the work-privacy conflict in German general practice personnel. Using baseline data of this study, the relationship between work-privacy conflict and job satisfaction (JS) was analyzed. In addition, factors associated with higher WPC were identified. Methods: At baseline, 366 participants (general practitioners (GPs) in leadership positions, employed general practitioners, and practice assistants) from 60 German practices completed a questionnaire addressing socio-demographic data and job characteristics. Standardized scales from the German version of the COPSOQ III requested data concerning job satisfaction and work-privacy conflict. Both scores range from 0 (lowest) to 100 (highest). Multilevel analysis accounted for the clustered data. Statistical analyses were performed using IBM SPSS and RStudio software, with a significance level set at *p* < 0.05. Results: Job satisfaction was 77.16 (mean value; SD = 14.30) among GPs in leadership positions (*n* = 84), 79.61 (SD = 12.85) in employed GPs (*n* = 28), and 72.58 (SD = 14.42) in practice assistants (*n* = 254). Mean values for the WPC-scale were higher for professionals with more responsibilities: GPs in leadership positions scored highest with 64.03 (SD = 29.96), followed by employed physicians (M = 45.54, SD =30.28), and practice assistants (M = 32.67, SD = 27.41). General practitioners and practice assistants working full-time reported significantly higher work-privacy conflict than those working part-time (*p* < 0.05). In a multilevel analysis, work-privacy conflict was significantly associated with job satisfaction (*p* < 0.001). A multiple regression analysis identified working hours, as well as and being a practice owner or an employed physician as factors significantly influencing WPC. Discussion: WPC was high among general practice leaders and practice personnel working full-time. Future interventions to support practice personnel should focus on reducing WPC, as there is good evidence of its effects on job satisfaction.

## 1. Introduction

In times where work environments are changing at an ever-faster pace, professional matters frequently conflict with employees’ private lives [1]. In the Copenhagen Psychosocial Questionnaire (COPSOQ), this role conflict is described as “work-privacy conflict” (WPC) [2]. Typical examples are private conflicts caused by long working hours, or having to leave work early due to child care issues or other life phase-specific needs [3]. Historically, WPC is related to research of Netemeyer et al. in 1996 [4], who examined the concept of work-family conflict with two directions: work interference with family (WIF) and family interference with work (FIW). Later, this concept was further developed: the term “work-life-conflict” came to include persons who do not live in traditional family units [5]. Garthus-Niegel et al. (2016) outlined that this term falsely suggests that work is separated from life and introduced the term “work-privacy conflict,” which most precisely describes potential role conflicts between professional and private lives [6]. All three constructs mentioned describe inter-role conflicts and are therefore closely related, allowing for comparisons between the studies [4].

Inter-role conflicts regarding work and private life are frequent among medical professionals: 44.3% of 7288 US physicians of all disciplines reported a work-home conflict, a construct similar to WPC, in the previous three weeks [7]. Physicians’ partners, who were surveyed parallelly, reported an even higher number of 55.7% [7]. Similarly, 47.8% of 543 physicians from German-speaking Switzerland suffered from a strong work-life conflict [8]. Among 296 German hospital physicians, the average WPC score was 74 (scale: 0 = lowest to 100 = highest on the German version of the COPSOQ), which differed significantly from a score of 45 for the German general working population [9]. According to a meta-analysis by Byron (2005) with over 60 articles, a high number of working hours is a risk factor for work-family conflict, while schedule flexibility is protective [3].

Spector defined job satisfaction (JS) as “the extent to which people like or dislike their jobs” [10]. According to a 2006 review, a high number of working hours, administrative burdens, unsatisfactory income, high workload, lack of time, and lack of recognition were negatively associated with the GPs’ job satisfaction [11]. Furthermore, a narrative review by Williams et al. outlined that poor job satisfaction in GPs is associated with more dissatisfied and less adherent patients [12]. Based on data from 676 German GPs and 2878 non-physician employees, Goetz et al. showed that non-physician practice staff rated their job satisfaction higher than GPs, except for the item “recognition for work” measured by the Warr–Cook–Wall questionnaire [13].

In several studies, low work-family conflict correlates with lower job stress [3] and lower burnout [14]. In reverse, high work-family conflict decreases job satisfaction [15,16,17]. In the field of medicine, for example, this interrelationship could be demonstrated for 351 Italian nurses [18] and 3535 Chinese physicians of all specialties [19]. Job satisfaction was studied in several GP populations [11,12,13]; there is already data measuring WPC among German hospital physicians [9]. Research of WPC and associations between WPC and job satisfaction in general practice personnel, stratified by occupational groups, is missing. Yet, such research is important for several reasons: first, primary care is secured mainly by the workforce of general practitioners and their teams [20]; second, data from hospital-based physicians cannot be extrapolated to German GPs, who predominantly own their practices, including all medical, financial, and administrative obligations. Therefore, we assume high WPC for practice owners following Garthus-Niegel et al., who described high WPC scores among self-employed individuals from a German random sample [6].

This study draws on baseline data from the IMPROVE*job* study, which is a prospective cluster-randomized controlled study to improve job satisfaction among GPs and practice personnel [21]. Here, we analyze the relationship between work-privacy conflict and job satisfaction in German general practitioners and practice assistants regarding work conditions.

## 2. Materials and Methods

### 2.1. Study Design

This study analyzes baseline data from the IMPROVE*job* study regarding the relationship between WPC and JS. Factors influencing WPC are also investigated. The details of the IMPROVE*job* study are described in the study protocol [21]; the socio-demographic data of the study population are published by Degen et al. [22].

In short, the publicly funded IMPROVE*job* study is a cluster-randomized controlled intervention trial (cRCT) with 60 general practices, 84 physicians in a leadership position, 28 employed physicians, and 254 practice assistants. The primary outcome is the change in job satisfaction after nine months. In addition, various secondary outcomes, such as WPC, quantitative, and emotional work demands were assessed. Participating GP practices were recruited from two university teaching practice networks and non-teaching practices from Germany’s North Rhine region. Study nurses collected baseline and follow-up data in the practices in a paper-pencil format. Every participant who completed the follow-up questionnaire received a monetary incentive [21]. The baseline data collection was completed in January 2020 before the COVID-19 pandemic.

### 2.2. Measurements

The following data are used for analysis:

Socio-demographic data: These comprise age (in years), gender (male/female/neutral), occupational group (practice owner, employed physician, practice assistant), marital status, persons in household over/under 18 years, and care for next-of-kin. The baseline results are published [22].

Work characteristics: The following aspects were assessed—working part-time vs. full-time and number of patients per quarter per practice.

Work-privacy conflict (WPC): WPC was measured using the respective scale of the 2018 German version of the international Copenhagen Psychosocial Questionnaire (COPSOQ III version) [2], which is a validated questionnaire for the measurement of psychosocial factors at work. The WPC scale of this instrument comprises two items (“The demands of my work interfere with my home and family life”; “The amount of time my job takes up makes it difficult to fulfil my family responsibilities”) and has a reliability of Cronbach’s alpha = 0.92 [2]. The response categories are as follows: strongly agree, somewhat agree, undecided, somewhat disagree, strongly disagree. Following the COPSOQ manual, these were transformed into a numerical scale from 0–100, with high values implying a strong WPC.

Job satisfaction was assessed using the respective scale of the COPSOQ III, which consists of six items: “Regarding your work in general: how pleased are you with: (1) your work prospects? (2) the people you work with? (3) the physical working conditions? (4) the way your group is run? (5) the way your abilities are used? (6) your job as a whole, everything taken into consideration.” Participants could choose between the following response categories: very satisfied, satisfied, neither/nor, unsatisfied, highly unsatisfied. Identical to the WPC scale, the answers were transformed into a numerical value from 0–100 and averaged, with high values implying high job satisfaction. Based on Nübling et al. [2]; internal consistency was Cronbach´s alpha = 0.79.

### 2.3. Statistical Analysis

Standard descriptive methods were applied to analyze all variables respecting their measurement level. Parametric measures, such as mean and standard deviation, are reported to allow for comparability of the results. The standard deviation is based on variance estimation considering the practice clusters. The COPSOQ Scales for WPC and JS were calculated following the respective manual. Analyses were performed for the whole sample and stratified according to professional group (practice owner, employed physician, practice assistant). Multilevel regression analysis was performed to describe associations between WPC and job satisfaction while respecting the clustered data structure. A hierarchical linear model was calculated to identify factors associated with high WPC, with respect for socio-demographic and work characteristics. The effect size is described by regression coefficients. SPSS Statistics 27 (IBM Cooperation, Armonk, Ny, USA, 2020) and RStudio software were used for statistical analyses. The significance level was set at *p* < 0.05.

## 3. Results

### 3.1. Descriptive Results and Demographic Characteristics

A total of 366 participants from 60 practices participated in the study: 112 GPs and GPs in training (84 practice owners, 28 employed physicians) and 254 practice assistants. The gender distribution showed a marked difference between the physicians and the practice assistants: 58.9% were female among the physicians, as were 99.6% of the practice assistants. The mean age of the owners was about 10 and 13.5 years higher, respectively, than that of employed physicians (54.3 vs. 44.8 years) and practice assistants (54.3 vs. 40.9 years). Practice assistants were more likely to work part-time than physicians (58.54% vs. 25%). Details on the socio-demographic characteristics for the total population, stratified by professional groups, are presented in Table 1, as presented in our prior publication [22].

### 3.2. Work-Privacy Conflict

**Figure 1:**
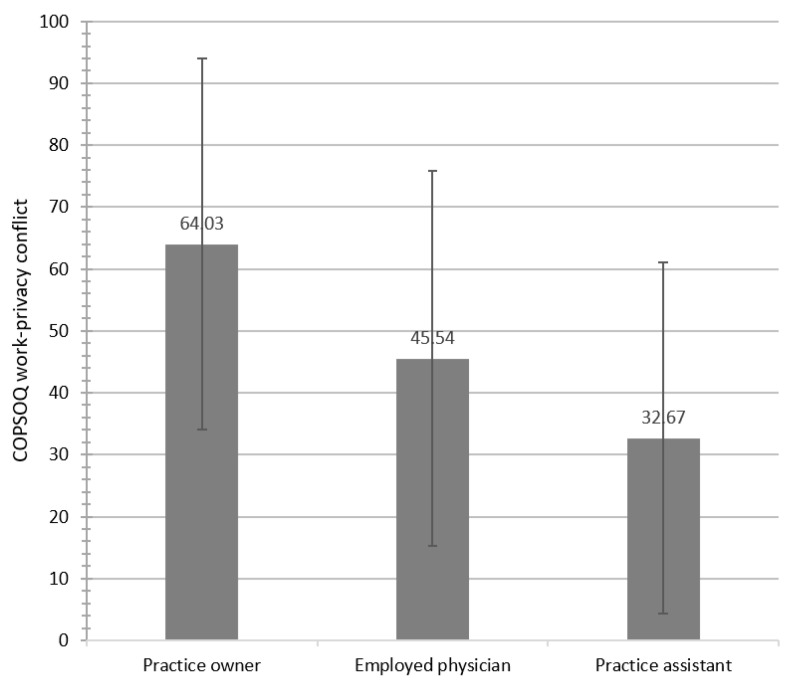
Overview of the COPSOQ work-privacy conflict scores by occupational group (High values imply a strong WPC).

Full-time workers reported higher WPC scores than colleagues working part-time, except employed physicians. Practice owners, employed physicians, and practice assistants differed significantly regarding WPC (F(2.361) = 35.31, *p* < 0.001). Post-hoc tests indicated statistically significant differences between the WPC means of the practice owners and employed physicians and, similarly, the practice owners and practice assistants. Male participants showed significantly higher mean WPC scores than their female colleagues (M = 59.84, SD = 30.82 vs. M = 37.88, SD = 30.64; T(362) = 4.55, *p* < 0.001). Female employed physicians had higher WPC scores than male employed physicians; sub analyses showed higher WPC among part-time working female physicians than males (female part-time: *n* = 18, WPC 49.55; male part-time: *n* = 2; WPC 12.50). For details, see Table 2 and Figure 1.

### 3.3. Work-Privacy Conflict and Job Satisfaction

The mean job satisfaction score was 77.16 (SD = 14.30) for GPs in a leadership position (*n* = 84), 79.61 (SD = 12.85) for employed GPs (*n* = 28), and 72.58 (SD = 14.42) for practice assistants (*n* = 254). A more comprehensive and detailed presentation of the descriptive JS scores can be found in Degen et al. [22]. In the multilevel analysis, work-privacy conflict was statistically significantly associated with job satisfaction respecting cluster effects (*b* = −0.10, *SE_b_* = 0.02, *t* = −4.29, *p* < 0.001). The negative regression coefficient implies that low WPC scores are associated with higher job satisfaction and reverse. The multilevel regression analysis showed that being a practice owner or employed physician and working full-time were significantly associated with increased WPC. For details, see Table 3.

## 4. Discussion

In our study, male and female practice owners showed significantly higher WPC scores than their employed colleagues and practice assistants. This finding aligns with results from the representative, population-based German Gutenberg Health Study of 3709 professionals, which showed higher WPC among academic self-employed professions (physician, attorney, tax consultant; mean WPC women: 43; men: 45) and managers (mean WPC women: 47; men: 45) [6]. However, using the same COPSOQ instrument, the average WPC scores were higher among the physician leaders we studied (total mean: 64.03; women: 62.35; men: 64.82). Our findings are consistent with data from hospital physicians, which showed higher scores on the work interfering with family conflict scale (WIF) than the general population (mean 74 vs. mean 45) [9]. Within our physician population, WPC differed markedly between practice owners and employed physicians (owners: 64.03; employed physicians: 45.54), which is explained by several factors, e.g., a higher workload, existential concerns, and ongoing management issues, in addition to regular patient care. These results align with the findings of Byron, which showed that hours spent at work and self-employment are predictors of work-family conflict [3]. The central role of the hours spent at work as a factor associated with WPC is confirmed in our regression analysis. Participants working full-time reported higher WPC than their colleagues working part-time. In our study, the average part-time hours of work across all occupational groups was 25.89 h/week. PAs who were employed full time worked an average of 40.21 h/week. Based on reliable data from the Central Institute for Statutory Health Insurance in Germany, practice owners reported an average workload of 49.3 h/week [23]. Higher WPC among professionals working full-time was also described in the studies of Byron [3] and Garthus-Niegel [6]. Interestingly, while there is only a small WPC difference between female and male practice owners, WPC was 18 points higher in female than male employed physicians. This is explained by higher WPC scores for part-time working female employed physicians. Their higher scores might be related to engagement in childcare to a larger extend than their male spouses, although this pattern is changing in Germany [24,25]. This finding is interesting, since WPC research has become more important, given a weakening of traditional gender roles [3]. However, our data show much higher WPC among female employed physicians working part-time than their male counterparts. This may reflect a stronger involvement of women in traditional family work.

In a large Canadian 2013 National Physician´s Survey, with more than 5000 family physician participants, 72% were satisfied with their professional lives, while 43.5% were not satisfied with their work-life balance [26]. Although our sample was much smaller, similar results were shown for German practice owners, with a JS score of 77.16 (mean COPSOQ reference population = 63.1 [27]) and a high WPC score of 64.03 (mean COPSOQ reference population = 39.0 [27]). In contrast, data from the German COPSOQ reference population with over 200,000 participants from various occupational groups showed much lower WPC and JS scores [27]. Since the practice owners were older on average, the question arises whether different work values between the generations are responsible for our results. This is consistent with the findings of Twenge et al., who found that younger generations place more value on leisure and extrinsic rewards than do older generations [28].

In the Canadian study, the factors associated with higher JS were a moderate number of working hours per week and having a special focus of interest in their practice [26]. In our sample, a high number of working hours was associated with WPC. Although prospective studies of job satisfaction among German primary care physicians are missing, the comparison with data published by Goetz et al. in 2011, based on 676 German GPs, indicates a rather stable situation: using the Warr–Cook–Wall questionnaire, a JS of 5.56 on a scale from 1 (extremely dissatisfied) to 7 (extremely satisfied) was shown [13].

## 5. Strengths and Limitations

The participation of complete practice teams, the diversity of practice workplaces, and the comprehensive data collection are the strengths of this study. However, the cross-sectional nature of the data analyzed does not allow for the analyses of predictors, while longitudinal data is needed to investigate changes over time and approaches for potential prevention.

## 6. Conclusions

A physicians’ job satisfaction influences patients’ satisfaction with care [12], as well as the availability of a strong primary care workforce [11,13]. As a theoretical implication of our study, future research should specifically address factors such as strategies to realize well-managed working hours, e.g., by optimizing work processes and the use of delegation models [29]. On a practical level, leadership training for GP practice owners with high WPC values should be implemented to decrease work-privacy conflict, to increase job satisfaction, and to prevent adverse outcomes such as emotional exhaustion [30], burnout [31], and low life satisfaction [32].

## Figures and Tables

**Table 1:** Socio-demographic description of participants (baseline), (published in Degen et al. [22]).

	Total Sample	Practice Owner	Employed Physician	Practice Assistant
Variable	N = 366	N = 84	N = 28	N = 254
Female, %	87.1	52.4	78.6	99.6
Age in years, mean (SD)	44.4 (12.8)	54.3 (6.2)	44.8 (9.8)	41.0 (13.0)
Years in current practice, mean (SD)	10.0 (9.1)	15.3 (8.4)	3.9 (5.4)	8.8 (8.9)
Working full time, %	52.0	90.5	28.6	41.5
Living in a relationship/ married, %	78.6	87.8	88.9	74.5
Persons in household over 18 years, mean (SD)	2.2 (1.0)	2.1 (1.0)	2.0 (0.5)	2.2 (1.1)
Persons in household under 18 years, mean (SD)	1.2 (1.0)	1.3 (1.3)	1.4 (1.0)	1.0 (0.9)
Care for next-of-kin, %	20.8	21.7	0.0	22.9
Professional characteristics of physicians (N = 112)				
Years since accreditation as physician, mean (SD)	24 (9.1)	26.6 (7.2)	16.3 (9.7)	-
Years since licensed for the statutory health insurance, mean (SD)	14.5 (9.4)	16.4 (8.4)	5.8 (8.8)	-
Physician in GP training, %	-	-	25.0	-
Professional characteristics of practice assistants (N = 254)				
Years since graduation, mean (SD)	-	-	-	19.9 (13.3)
Qualification as practice assistant, %	-	-	-	81.9
No additional qualification, %	-	-	-	64.2
Practice assistant in training, %	-	-	-	7.5
Average working hours in last 3 months per week, mean (SD)	-	-	-	32.7 (10.7)

**Table 2:** Work-privacy conflict (WPC) scores stratified by various socio-demographic and professional characteristics.

	Mean	SD	Min.	Max.
**Occupational group**				
Practice owner	64.03	29.96	0	100
male	64.82	27.79	0	100
female	62.35	31.75	0	100
Employed physician *	45.54	30.28	0	100
male	31.25	38.53	0	100
female	49.43	27.41	0	100
Practice assistant **	32.67	28.35	0	100
**Gender**				
m	59.84	30.82	0	100
f	37.88	30.64	0	100
**Age (years)**				
<20–29	33.74	28.46	0	100
30–49	37.85	28.82	0	100
50–69	44.62	34.17	0	100
**Working part-time**				
Total	31.30	27.69	0	100
Practice owner *	43.75	29.12	0	87.50
Employed physician *	45.63	29.88	0	100
Practice assistant	28.53	26.71	0	100
**Working full-time**				
Total	51.05	31.60	0	100
Practice owner	66.17	29.52	0	100
Employed physician *	45.31	33.37	0	100
Practice assistant	39.74	28.94	0	100
**Marital status**				
Living alone	40.18	31.75	0	100
In a relationship or married	41.47	31.48	0	100
**Taking care of relatives?**				
Yes	41.52	32.37	0	100
No	40.57	31.30	0	100

Annotations. * Low case number; model fit does not converge. Values are reported without cluster adjustment. ** Only highly aggregated values are reported in order to maintain identity (low number of male PAs).

**Table 3:** Multilevel regression analysis: associations between WPC and socio-demographic, work, and practice characteristics.

	Work-Privacy Conflict		
	*b*	*SE_b_*	*t*
Age	0.13	0.24	1.18
Gender ^a^	1.74	7.78	0.22
Marital status ^b^	2.71	5.80	0.47
Persons in household over 18 years	0.72	2.04	0.53
Persons in household under 18 years	−0.91	2.05	−0.44
Care for next-of-kin ^c^	3.87	5.41	0.71
Occupational group: Practice owner ^d^	20.22	8.20	2.47 *
Occupational group: Employed physician ^d^	17.98	7.17	2.51 *
Working part-time/full-time ^e^	12.15	5.61	2.17 *
Patients per quarter per practice	−0.34	1.00	−0.34

Annotations. * *p* < 0.05, *b*—regression coefficient b; *SE_b_*—standard error; *t*—*t*-value; ^a^ coded as: 0 = male, 1 = female; ^b^: 0 = living alone, 1 = relationship or married; ^c^: 0 = no, 1 = yes; ^d^ Dummy variables for occupational group: 0 = no practice owner/employed physician, 1 = practice owner/employed physician; regression coefficient b shows the expected difference in relation to the reference category practice assistant; ^e^: working part-time = 0, working full-time = 1.

## Data Availability

There are no plans to grant access to full protocol, participant-level datasets, or statistical codes, as data contain potentially identifying information.
